# Dissecting the disconnect between circuit activation and dominant adaptive evolution in cytoplasmic phage-assisted continuous evolution (PACE) of an EGFR nanobody

**DOI:** 10.3389/fbioe.2026.1829373

**Published:** 2026-05-29

**Authors:** Jie-Ning Chuang, Jacob Purcell, Loki Sangalli, Joseph Rosenbluh, Gavin J. Knott, Simon Corrie, Gil Garnier

**Affiliations:** 1 Bioresource Processing Research Institute of Australia (BioPRIA), Department of Chemical and Biological Engineering, Monash University, Clayton, VIC, Australia; 2 Department of Biochemistry and Molecular Biology, Biomedicine Discovery Institute, Monash University, Clayton, VIC, Australia; 3 AI Protein Design Program, Biomedicine Discovery Institute, Monash University, Clayton, VIC, Australia

**Keywords:** cytoplasm, epidermal growth factor (EGFR), nanobody, phage-assisted continuous evolution (PACE), protein-protein interaction

## Abstract

**Introduction:**

Cytoplasmic phage-assisted continuous evolution (PACE) is widely used due to its low selection pressure and straightforward circuit design. However, the reducing environment limits its applicability to mammalian proteins that rely on disulfide bonds or glycosylation.

**Methods:**

To evaluate whether these biochemical constraints inherently hinder cytoplasmic evolution, we confined both the antibody and the target protein to single-domain formats. We examined circuit activation and evolution of a 7D12 nanobody targeting the epidermal growth factor receptor domain III (EGFR DIII).

**Results:**

Nanobodies and EGFR DIII expressed in the cytoplasm retained their soluble fractions and pairing activity. These enabled efficient activation of the two-hybrid circuit and robust phage propagation. Despite successful circuit activation, neither non-continuous flow (PANCE) nor PACE generated affinity-enhanced variants under extended drift conditions and graded selection pressures designed to increase mutational diversity.

**Discussion:**

Although optimal PACE operating conditions inhibited cheater gene recombination and enabled sustained POI mutagenesis, mutational convergence may be more sensitive to organismal incompatibility than circuit activation. This aligns with the observed decoupling between circuit activation and productive adaptive evolution. Moreover, structural analysis and predicted saturation mutagenesis at the binding interface are consistent with interface accessibility as a plausible constraint. The 7D12 nanobody contains a narrow, protruding CDR3, and most substitutions at the CDR1 -CDR3 interface are neutral or deleterious. Although single-domain mammalian proteins can overcome organismal incompatibility for cytoplasmic PACE circuit activation, their evolvability may be influenced by limitations in the accessibility of beneficial mutations within the binding interface and by their combination with organism-level factors. These results support a multifactorial basis for evolvability in cytoplasmic PACE, involving both interface accessibility and system-level factors.

## Graphical Abstract

**Figure:**
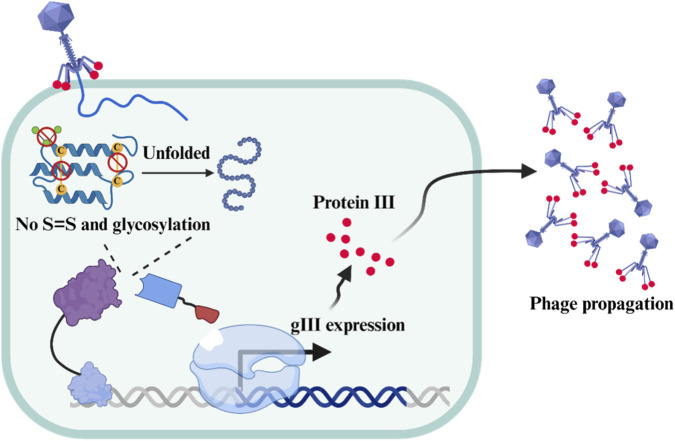

## Highlights

- Cytoplasmic PACE allows sufficient circuit activation for mammalian protein interactions despite the loss of disulfide bonds and glycosylations.
- Lower selection stringency allows phage to escape circuit selection via cheater gIII recombination.
- Continuous flow is critical to prohibit cheater gIII recombination on the phage genome.
- Circuit activation and evolvability represent mechanistically distinct requirements in mammalian protein PACE.
- Restricted mutational accessibility at the binding interface may limit evolutionary outcomes.

## 1 Introduction

Diagnostic and therapeutic development requires affinity modulation to improve target labelling and sensitivity due to the rapid pathogen and virus mutagenesis (Landry et al., 2012; Rudnick et al., 2011; Opaliński et al., 2018; Macdonald et al., 2022). Although crafting efficient approaches for high affinity has been ongoing in antibody engineering, the current library-driven processes still require separate and iterative steps involving randomized mutagenesis, expression, and screening, with no guarantees of success. To simplify this labor intensive involvement, several strategies were explored to introduce mutations directly on target plasmids *in vivo*. EvolvR enables gene editing in the *E. coli* system with a CRISPR-guided nickase which introduces mutagenesis via epPol I (Halperin et al., 2018; Tou et al., 2020; Hurtado et al., 2025). A similar strategy was tested with a mammalian cell system by substituting epPol I with an activation-induced cytidine deaminase (AID) (Hess et al., 2016; Berríos et al., 2024). As a result, the applicability of directed editing complex can be extended to proteins with a sophisticated combination of intra-molecular disulfide bonds and glycosylated residues. Of those, OrthoRep from yeasts is a compromise which retains the benefits of accurate folding while exhibiting short growth cycles (Ravikumar et al., 2014; Rix et al., 2020; Ravikumar et al., 2018; Rix et al., 2024). This technique only orthogonally mutates specific linear plasmids; it can therefore be integrated with a display system to reduce the workload during expression, purification and screening. Although those in vivo-directed evolution strategies promote continuous evolution for the protein of interest (POI), frequent *in vitro* interventions still require significant lab work and time to screen desired mutations on the POI.

**FIGURE 1:**
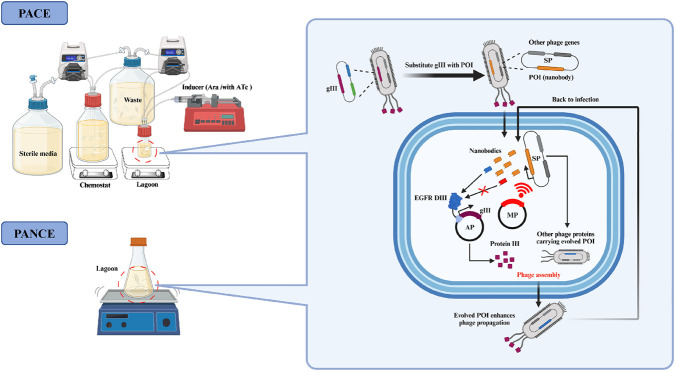
Schematic of the PACE and PANCE systems using a two-hybrid circuit. During PACE, sterile 2xYT media was continuously primed into the chemostat for host cell culture. When the OD_600_ reached 0.4–0.6, the cells were supplied to the lagoon and were exposed to phage. As for the PANCE, the entire selection process was conducted by transferring the purified phage to a dedicated infection/incubation flask. The gIII (purple) of phage was replaced by the POI (orange) to link protein interaction to the phage life cycle. The host cells were co-transformed with AP carrying the target protein and MP carrying the mutagenesis gene. During infection, POI with improved affinity aims to enhance gIII expression and subsequent phage propagation. The resulting phage is predominantly expected to carry POI with improved affinity (blue). The affinity of POI can be further optimized through multiple rounds of infection.

To achieve autonomous protein mutation and binding screening *in vivo*, Liu’s group proposed a two-hybrid circuit for phage-assisted continuous evolution (PACE) (Bardran et al., 2016). The critical linkage involves replacing gIII of the phage genome with POI-rpoZ (RNAP ω subunit) fusion. When the POI-rpoZ fusion engages with the target protein fused to the 434 cI repressor, a complex is formed and located at the cognate sequence encoded upstream of gIII. This guides the reconstitution of the subunit onto RNAP. The intact RNAP allows the expected gIII transcription and subsequent pIII expression for phage infectivity. The remaining genotypes in the lagoon only gather those that enable enhanced protein-protein interaction to support robust phage propagation under the given selection pressure in the continuous flow (PACE) or non-continuous flow (PANCE) systems, as illustrated in Figure 1. While the two-hybrid circuit has been validated across many proteins, almost all examples constrain this circuit to disulfide and glycosylation-free proteins and domains (Wang et al., 2018; Pu et al., 2018; Zhou et al., 2024; Styles et al., 2025). This is because the bacterial cellular environments are not compatible with the evolution of mammalian protein interactions (Castillo-Corujo et al., 2024; Lobstein et al., 2012). This is a challenge for applying PACE to antibody engineering, particularly for antibodies that rely on multiple intramolecular disulfide bonds and glycosylated residues to stabilize the folded structure and interface geometry for preserving binding capability.

Exporting the POI to the periplasm (oxidizing environment) is another approach to mitigate effects on protein core folding (Liu et al., 2012; Matos et al., 2014). However, this process requires tedious optimization with multiple stringent selection steps, including intein-mediated periplasmic export, scFv dimerization, and the lack of guidance for antigen engagement. Even with optimal components of the PACE circuit (Morrison et al., 2021), the POI-peptide interaction in the periplasmic space narrowed the mutational diversity of the evolutionary landscape. These issues have motivated the deployment of simple-structure proteins in the cytoplasmic PACE to mitigate the effects of unfolded proteins. A recent study by Ye et al. (Ye et al., 2020) demonstrated that cytoplasmic PACE could enhance the protein affinity of humanized PD1. In that work, PD1 contained extended structural features, such as a signal peptide and flexible terminal regions. Dominant adaptive mutations started to occur primarily in these peripheral regions rather than at the core binding interface; meanwhile a new binding interface was established at the opposite β-sheet. Although this implies that deploying single-domain proteins with fewer intradisulfide bridges and glycosylated residues in the cytoplasmic PACE may alleviate biochemical incompatibilities, whether such simplification is sufficient to support productive adaptive evolution on the original interface remains unclear for mammalian proteins with complex interaction interfaces.

Blocking the epidermal growth factor receptor domain III (EGFR DIII) is critical for suppressing EGF-induced tumor cell proliferation in many solid tumors (Yewale et al., 2013; Zanetti-Domingues et al., 2018; Iyer et al., 2024; Sigismund et al., 2018; Uribe et al., 2021; Ciardiello and Tortora, 2008). In this study, we confined the EGFR antibody (7D12 nanobody) (Schmitz et al., 2013) and EGFR (EGFR DIII) to a single-domain format to evaluate PACE circuit activation and evolution in the bacterial cytoplasm. We used the same plasmids in the two-hybrid circuit, as these components have been optimized for maximum gene-level activation (Bardran et al., 2016). Even without stabilization by multiple intra-disulfide bridges and glycosylated residues, the 7D12-EGFR DIII interaction specifically activates RNAP reconstitution and subsequent gIII expression for phage propagation. However, no dominant adaptive mutations emerged despite robust circuit activity and a prolonged drift period. We systematically evaluated potential confounding factors, including cheater gene recombination, weak circuit activity, and technical issues with the materials and reactor setup. These observations, together with the strong correlation between binding interface and evolutionary outcomes (Morrison et al., 2021; Ye et al., 2020), motivated us to consider previously underappreciated interface-level determinants as potential contributors alongside other possible constraints. Structural analysis suggested that the narrow CDR3 (complementarity-determining region 3)-dominated interface on the nanobody may limit mutational accessibility. Moreover, saturation mutagenesis scanning further indicated that most amino acid substitutions within CDR1 and CDR3 are neutral or deleterious. These findings collectively supported a distinction between circuit activatability and evolvability in cytoplasmic PACE. Although the 7D12–EGFR DIII interaction enabled sufficient compatibility to activate the two-hybrid circuit and subsequent phage propagation, successful circuit activation alone does not guarantee dominant adaptive evolution. Our results are consistent with a model in which binding-interface geometry may contribute to evolutionary trajectories of mammalian protein interactions in cytoplasmic PACE. This factor may act in combination with other contributing factors to influence the observed evolutionary outcome.

## 2 Experimental section

All chemicals were purchased from Sigma-Aldrich unless otherwise specified.

### 2.1 Plasmid cloning and transformation

Among the different EGFR nanobodies (Schmitz et al., 2013; Sharifi et al., 2021; Tripathy and Pande, 2024), we chose the 7D12 nanobody because it can solely inhibit EGFR DIII. The protein sequences of 7D12 and EGFR DIII were obtained from PDB: 4KRL (Schmitz et al., 2013). GenScript Biotech Corporation synthesized 7D12 (WT nanobody), R30A (low-affinity nanobody), and EGFR DIII DNA fragments and optimized codons. Both nanobodies and EGFR DIII were individually cloned into the pET-28b (+) (Merck, Cat# 69865–3) and pET-15 (Merck, Cat# 69661–3) vectors through the digestion of NcoI (NEB #R3193) and XhoI (NEB #R0146) and subsequent ligation using high-fidelity (HIFI) assembly master mix (NEB #E2621). The optimal DNA molar ratio and incubation parameters are documented as per the manufacturer’s instructions. To construct our phages encoded with 7D12 and R30A nanobodies, we initially cloned nanobody-rpoZ fusions into pBT100.106b (addgene, Cat# 122601) using SapI restriction sites, followed by assembling with pBT13-splitC (addgene, Cat# 122596) and pBT13-splitD (addgene, Cat# 122597) via Golden Gate assembly (Thuronyi et al., 2019). The orientation of fusion was designed with a nanobody before the rpoZ subunit to avoid the disruption of nanobody/EGFR DIII pairing. For the accessory plasmid (AP), EGFR DIII was cloned at the ApaI and XbaI of pB092Aa (addgene, Cat# 79217) (Bardran et al., 2016).

The transformation was carried out by subjecting the ligation mixture to DH5α competent *E. coli* (NEB #C2987H) via heat shock. First, the competent cell stock was thawed on ice for 10 min, and 2 μL of ligation mixture was added to it. After incubation on ice for 30 min, the heat shock was performed at 42 °C for 30 s, followed by incubation on ice for 5 min to encapsulate the plasmid in the bacteria. The mixture was then supplemented with 900 μL of SOC medium (NEB #B9020S) and incubated in a shaking incubator at 200 rpm and 37 °C for 1 h. Finally, a 100 μL mixture was spread on a lysogeny broth (LB) agar plate supplemented with the corresponding antibiotics and incubated overnight at 37 °C. The resulting expression plasmids and AP were then transformed to BL21 (DE3) Competent *E. coli* (NEB #C2527) and s2208 cells, respectively. The s2208 cells are made of s2060 (addgene, Cat# 105064) carrying plasmid pJC175e (addgene, Cat# 79219) (Bardran et al., 2016). The SP transformation skipped the recovery step and was directly transferred to 2xYT broth (Invitrogen™) for further plaque assay analysis.

### 2.2 Protein expression and purification

BL21 (DE3) cells containing plasmid 7D12:pET-28b, R30A: pET-28b, or EGFR DIII_pET-15b were added into 5 mL LB broth and supplemented with the corresponding antibiotics for preculture preparation at 200 rpm shaking speed and 30 °C. The next day, 1 mL of overnight preculture was transferred to 100 mL of Terrific Broth (TB) medium supplemented with appropriate antibiotics for culture expansion until an OD_600_ reached 0.4–0.8. IPTG was spiked to a final concentration of 1 mM at this time point. After 24 h of induction, cells were harvested with centrifugation for 15 min at 6,000 g and 4 °C, and stored at −20 °C after discarding the supernatant.

Every 200 mg of cells was resuspended in 1 mL binding buffer (30 mM Tris-HCl, 300 mM NaCl, and 10 mM imidazole) supplemented with 0.1 mM PMSF, 1 mM benzamidine, 25 U/mL Turbonuclease, and 5% glycerol (Lobstein et al., 2012). After cell lysis via mechanical sonication, the cell debris could be removed by centrifugation at 10,000 x g and 4 °C for 20 min. The nanobodies and EGFR DIII were first purified through fast protein liquid chromatography (FPLC) using a HisTrap excel column (Cytiva). The purification steps were the same as per the manufacturer’s instructions. The elution was carried out with an additional 400 mM imidazole in the binding buffer. Amicon® ultra centrifugal filters were used for buffer exchange and removal of high-molecular-weight impurities. Finally, the protein composition was analyzed via SDS-PAGE using a pre-made gel (NuPAGE™ Bis-Tris Mini Protein Gels, Thermo Fisher Scientific) under electrophoresis at 150 V for 15 min.

### 2.3 Biolayer interferometry (BLI)

The determination of nanobody-antigen affinity was conducted using BLI which is an octet family platform from ForteBio, Pall Inc., as per the manufacturer’s protocol. The AR2G biosensor (Sartorius Australia Pty. Ltd.) was specifically adopted for determining the binding kinetics of our proteins. The carboxylic-terminated surface allows nanobodies to covalently immobilize on the probe via EDC/NHS coupling, providing a stable, reliable signal throughout the experiment. This process is pH-sensitive, so the nanobody solution for probe immobilization was typically prepared with 10 mM sodium acetate (pH 5.0) at a final concentration of 50 μg/mL. Sensor loading conditions were kept consistent across measurements to ensure comparable signal levels. Different from nanobodies, antigen dilutions were performed in 1x PBSK buffer (prepared by diluting 10x kinetics buffer with 1x PBS buffer). The association and dissociation curves were analyzed using the BLI built-in tool to derive the global *K*_*D*_ using a 1:1 Langmuir binding model, with no sensor regeneration between runs. Reference subtraction was applied to correct for background and non-specific signals. *K*_*D*_ values were obtained from a single measurement rather than multiple independent biological replicates.

### 2.4 Plaque assay

Plaque assay analysis was carried out to quantify phage titers. Each assay included a negative control to assess whether host cells were contaminated and four serial dilutions of the target phage to obtain countable plaques for titer determination. All plaque assays were conducted as single measurements; therefore, the reported titers represent representative values rather than statistically averaged measurements.

First, s2208 cells were grown in 2× YT liquid media supplemented with 100 μg/mL ampicillin at 37 °C until OD_600_ = 0.6–0.9 to prepare the preculture. To prepare the top-layered agar for the plaque assay, 750 μL fresh s2208 host cell culture, 120 μL Xgal, and 5 mL top 2x YT agar (a mixture of 20 mL 1.5% 2x YT agar with 30 mL 2x YT media) were mixed with 10 μL of four phage dilutions (100-fold). Finally, the mixtures were spread onto a 1.5% 2x YT agar plate supplemented with 100 μg/mL ampicillin, and the plates were incubated at 37 °C overnight. The phage titer can be calculated based on this formula: Titer (in pfu/mL) = (number of plaques) × (dilution factor) × 100 (Miller et al., 2020). Serial dilutions were used to ensure plaque counts fell within a countable range, providing internal consistency within each assay.

### 2.5 PACE and PANCE

The PANCE and PACE processes have been well documented by Miller et al. (Miller et al., 2020). The AP cells containing mutagenesis or drift plasmid (MP or DP), supplemented with appropriate antibiotics, were inoculated into 30 mL of 2xYT broth to obtain an overnight culture with an OD_600_ of around 0.4–0.8. This culture was transferred to the chemostat, where the flow rate of fresh media was maintained at a dilution rate of 1 V/h. Once the OD_600_ of the chemostat remained at 0.4–0.6, AP cells were dispensed to the lagoon at 1 V/h of dilution rate and supplemented with arabinose (Ara) (or anhyrotetracycline (ATc) for DP) at 0.6 mL/h until the OD_600_ was stable again. The ATc and Ara concentrations were selected based on the concentration thresholds reported by Miller et al. (Miller et al., 2020) to balance mutant pool accumulation and selection pressure. Purified phage (SP) was subjected to the lagoon at the final titer of 1 × 10^7^ pfu/mL. The evolved phage was sampled at a specific time interval. The single mutated phage was isolated via plaque assays. Phage propagation, plaque counts, and titer measurements were performed as representative observations without independent biological replication. They were primarily used to track trends in phage propagation and mutation emergence rather than to provide statistically powered comparisons. Each sampling round collected 10 plaques for sequencing. Rapid enrichment of dominant adaptive mutations typically improves fitness and leads to convergent genotypes within the phage population. With this idea in mind, sampling strategies commonly focus on identifying dominant variants that emerge during periods of increased phage propagation rather than exhaustively or randomly surveying low-frequency mutations. The PANCE followed a similar protocol in a batch system; therefore, the phage had to be transferred between different passages to achieve stepwise evolution.

### 2.6 Site-saturation scanning and indirect ELISA verification

The amino acids on CDR1 and CDR3 were identified from SAbDab (Dunbar et al., 2014) and processed for site-saturation scanning using mCSM-AB2 (Myung et al., 2020). We then designed five mutants using the ΔΔG values calculated from Equations 1, 2, each with single or multiple mutations. The binding strength should align with the predictions.

$$\Delta G=RTln\left(K_{D}\right)$$ (1)

$$\Delta \Delta G=\Delta G_{wild}-\Delta G_{mut}$$ (2)

Nanobody cloning, protein expression, and purification were the same as described in Section 2.1, but the expression was conducted using SHuffle cells (NEB, Cat# C3026) to stabilize intramolecular disulfide bond formation. For the indirect ELISA, we first coated EGFR DIII onto the 96-well plate, then blocked the active sites with bovine serum albumin (BSA). After incubation with the WT nanobody or other mutants, we probed the samples with anti-VHH-HRP conjugates (Genscript, Cat# A01861-200) and measured absorbance at 565 nm. The experiments include a negative control with EGFR DIII and BSA alone, and another control without any nanobody to assess non-specific binding between EGFR DIII and anti-VHH-HRP conjugates.

## 3 Results

### 3.1 PACE circuit activity and specificity

**FIGURE 2:**
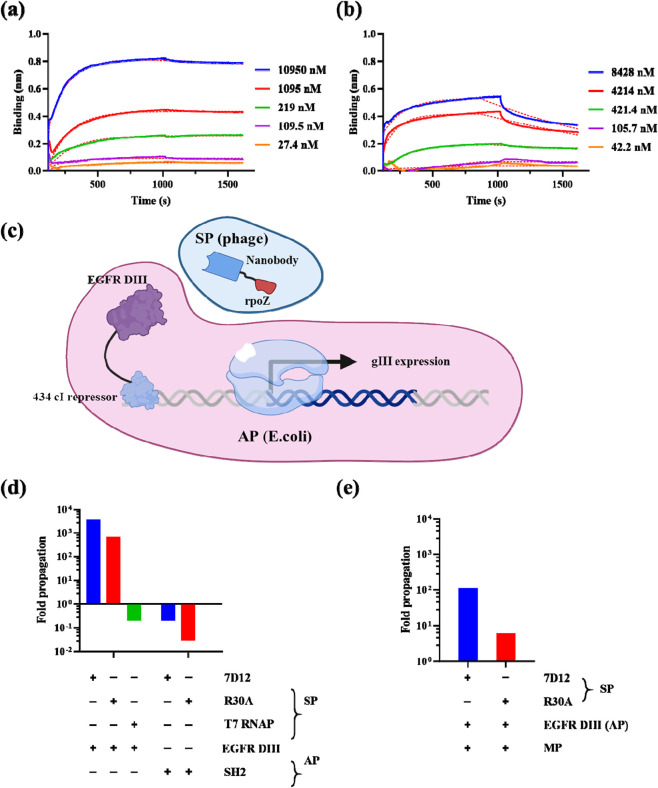
Nanobody-antigen affinity measurement by BLI. Kinetic analysis of **(a)** wild-type (7D12) and **(b)** weak-affinity (R30A) anti-EGFR nanobodies associated and dissociated with a series of EGFR DIII dilutions. PACE **(c)** circuit and its activity and specificity analysis via plaque assay with different plasmid combinations: **(d)** SP + AP; **(e)** SP + AP + MP.

The 7D12 nanobody was chosen to build into the selection plasmid (SP). We also introduce the R30A mutation to obtain a variant with lower affinity. This allows us to observe the preference for an evolutionary trajectory, either affinity restoration or toward different mutations. The *in vitro* expression was conducted using BL21 (DE3) to simulate the protein folding in the reducing cytoplasm. SDS-PAGE in Supplementary Figure S1 shows a clear single band for 7D12 and R30A nanobodies at around 14 kDa as well as EGFR DIII at 24 kDa. We implemented a range of EGFR DIII dilutions to determine the dissociation constants (*K*_*D*_) of 7D12 (wild-type) and R30A (weak-affinity mutant) by BLI, as illustrated in Figures 2a,b. 7D12 generally showed a higher overall signal than R30A at similar EGFR DIII dilutions. This phenomenon was further validated through dynamic analysis using the BLI built-in tool. The *K*_*D*_ of 7D12 is 47 nM and that of R30A is 149 nM. The kinetic parameters k_on_ and k_off_ are also documented in Supplementary Table S1. The parameter summary substantiated our observation of robust binding intensities for 7D12 and significant dissociation for R30A.

The PACE circuit construct in this study is shown in Figure 2c. Its activity and selectivity used for evolving anti-EGFR nanobody were validated using activity-dependent plaque assays. Figure 2d depicts the titer change when AP (EGFR DIII) and non-AP (SH2) cells are infected by phage-carrying 7D12 or R30A nanobodies. The phage-carrying T7 RNA polymerase was used to confirm whether gIII leaks in our circuit design. The results show that the interaction between phages carrying 7D12 or R30A nanobodies and AP cells carrying EGFR DIII can generate significant phage proliferation compared to the AP cells carrying SH2 antigens or phages carrying T7 RNA polymerase. This is because accurate protein-protein interactions lead to gIII expression, which supports phage propagation. When we co-transformed MP into AP cells to confirm whether our circuit design possesses minimum activity for initiating PACE, the titer propagation of 7D12 and R30A phage decreases from 600-3000-fold to 10-100-fold after 20 h of culture (Figure 2e). Although undesired mutations made phage survival even harsher, the 7D12 phage fold propagation still reached the minimum activity required (more than 50-fold (Miller et al., 2020)) to initiate PACE.

### 3.2 Recombinant gIII on the phage after PANCE

**FIGURE 3:**
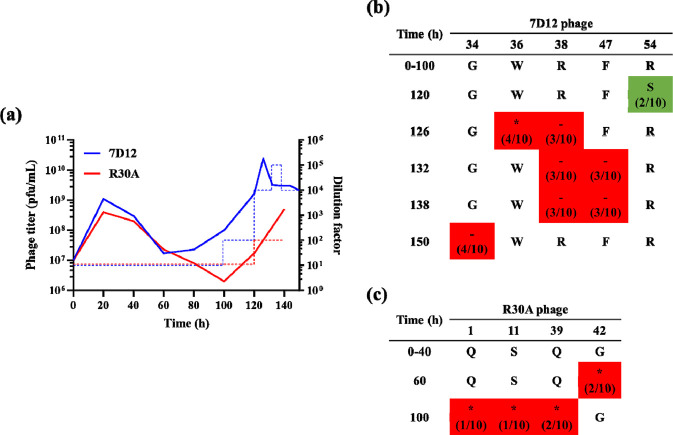
**(a)** Phage titers and the corresponding dilution rates for phage carrying 7D12 and R30A nanobody over the period of PANCE. **(b,c)** Consensus mutations identified in 7D12 and R30A phage during PANCE. Numbers in parentheses represent the frequency of each mutation observed in ten plaques. Mutations leading to amino acid substitution are colored in green, and those leading to deletions or stop codons are highlighted in red.

The mutagenesis plasmid (MP) was found to be toxic to circuit activation owing to undesired mutagenesis. To address this issue, phage-assisted non-continuous evolution (PANCE) was adopted to maintain phage survival by mitigating selection stringency (Doman et al., 2023; Zhang et al., 2024). The titers 7D12 and R30A phage over the passages versus dilution rates are illustrated in Figure 3a. The 7D12 and R30A phage titers rapidly climbed from 80 to 100 h, respectively. It was expected that consensus mutations would start occurring, followed by converged differences. Therefore, we picked ten plaques from specific rounds for sequencing and the results are listed in Figures 3b,c. The first consensus mutation of 7D12 PANCE is R54S, which emerged in two plaques at 120 h. However, several amino acid deletions became dominant at R38, G34, and F47 from 126 to 150 h. Similar results happened on R30A PANCE starting from the third passage, but in another way: converting the original codon to a stop codon.

**FIGURE 4:**
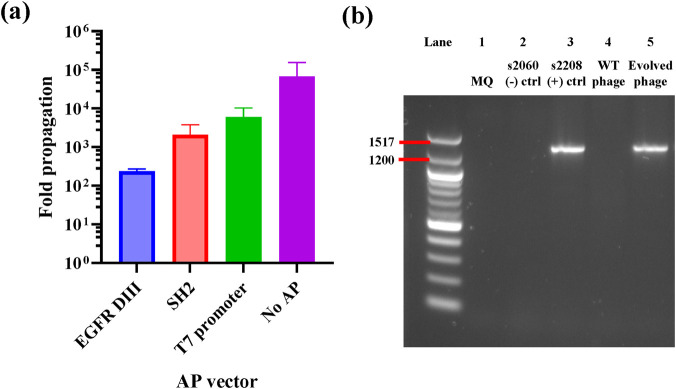
**(a)** Fold propagation after evolved phage upon infection of host cells carrying EGFR DIII, SH2, T7 promoter only, or no insert. Each value represents the mean ± SD from two independent replicates. **(b)** Amplification of gIII from MQ water, s2060 and s2208 cells, and WT and evolved phages.

To better understand the reason behind this, we replicated the evolved phage/PACE cell infection with four AP cell combinations: (1) EGFR DIII, (2) SH2, (3) T7 promoter, and (4) blank cells. All AP cells surprisingly demonstrated robust propagation ranging from 200-fold to 130,000-fold in two replicates, as shown in Figure 4a. Given that the T7 promoter AP cell lacked RNA polymerase, the circuit activated by truncated nanobodies was excluded. The gIII recombination in evolved phages was the only mechanism capable of readily generating protein III. We then performed PCR to confirm whether gIII exists in double-distilled water, blank s2060 cells, s2208 cells, and WT and evolved phages (Figure 4b). Double-distilled water, s2060 cells, and WT phages are negative controls; therefore, no band was detected in lane 1, 2, and 4. The evolved phage exhibited a bright band at the same size as that of s2208 cells (positive control), which explains why evolved phages could escape the circuit selection and activate any AP cells due to cheater gIII expression.

### 3.3 Phage titer collapsed after PACE

**FIGURE 5:**
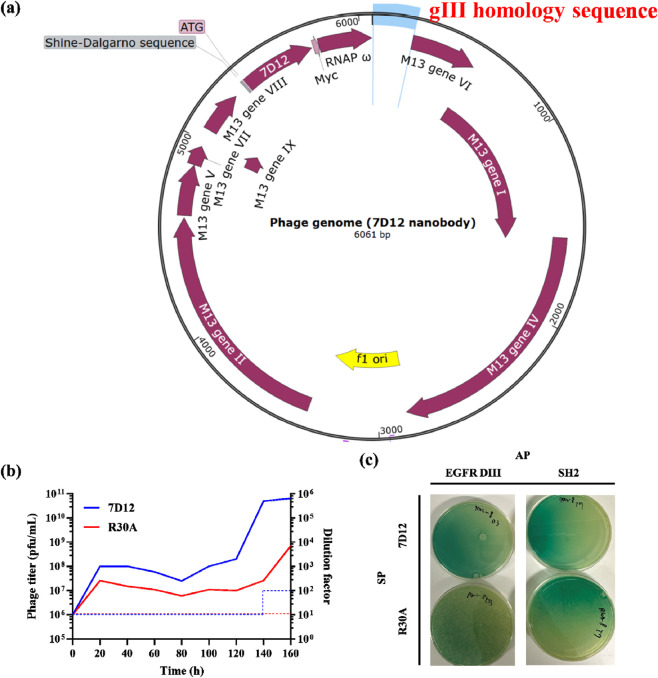
**(a)** Schematic illustration of the gIII homology region on the phage genome. **(b)** Phage titers and their dilution rates for 7D12 and R30A phage across 160 h of PANCE. **(c)** Activity analysis of evolved 7D12 and R30A phages using plaque assays on AP cells carrying EGFR DIII and SH2. All evolved phages were applied without dilutions.

Such unexpected gIII recombination can be attributed either to the presence of gIII homology sequences in the original phages (Figure 5a) or to the accumulation of mutations in the same phage molecule. We first utilized Golden Gate assembly to reclone 7D12 and R30A phage with updated vectors (Addgene#: 138,521 and 138,523) for recombinant gIII (Zhang et al., 2024), followed by 160 h of PANCE. The detailed deletions or insertions are shown in Supplementary Figure S2. The phage titers and dilution rates throughout PANCE are shown in Figure 5b. The phage titers of 7D12 and R30A remained similar to the initial levels and then began a steep increase at 120 and 140 h, respectively. The slower increase in R30A phage titer compared to that of 7D12 phage is due to a lower affinity to activate the two-hybrid circuit. The titer rise with gIII homology-removed 7D12 and R30A phage required an extra 40 h over their counterparts carrying gIII homology sequences. This suggests that removing the gIII homology part could be critical to inhibiting gIII recombination. We then directly plated the phage harvested at 160 h onto target AP cells (EGFR DIII) to isolate the active phage for later assays to determine whether the evolved phage carried intact gIII. The assay results with inoculating AP (EGFR DIII) and non-AP (SH2) cells are shown in Figure 5c. Evolved 7D12 and R30A phages are highly infectious and can activate both circuits. Although the removal of gIII homology successfully delayed recombination, gIII restoration could still be observed over extended MP interference. This strategy failed to support our nanobody evolution in a way that continuously enhanced affinity.

Kinetic flow is an alternative strategy to prevent the same phage molecule accumulating undesired mutations from weak selection pressure. Figure 2d indicates that our circuit activity might be insufficient to sustain phage propagation under continuous dilution during PACE. To exclude problems related to DP or MP preparation or reactor setup, we conducted a control experiment using a standard circuit to evolve T7 RNA polymerase for T3 promoter recognition (Supplementary Figure S3a). This process consisted of a 3 h drift with 100 mg/mL ATc for the accumulation of randomized mutations, a 3 h drift with 10 mg/mL ATc for the removal of scatter mutations, and a final 48 h selection without ATc supplement for the convergence of active mutations. The phage titer and dilution factor over time are recorded in Supplementary Figure S3b. Since T7 RNA polymerase could not initially be activated by the T3 promoter, the phage titer dropped drastically in the first 6 h, followed by subsequent phage titer spiking due to the appearance of active mutations. The plaque assays were conducted to confirm whether the evolved phage was active and gIII recombinant, as shown in Supplementary Figure S3c. The blue plaques only existed on the AP cell carrying the T3 promoter after the ATc drift. This confirms that the mutations are converged and no gIII is encoded on the genome. Sanger sequencing results identified three consensus mutations from three plaques harvested after 48 h of PACE, namely R34P, M219R, and I244V. Among those, M219R is the critical mutation that enables T7 RNA polymerase to identify the T3 promoter as reported by Esvelt et al. (2011).

**FIGURE 6:**
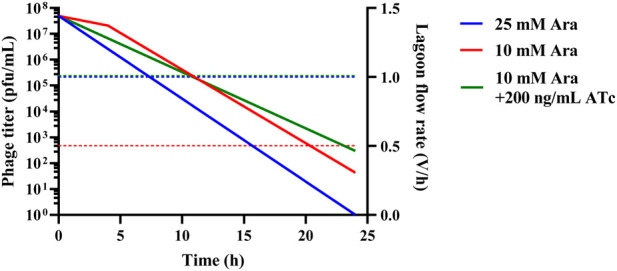
7D12 phage titer and lagoon flow rate over time under different supplementation strategies: (1) 25 mM arabinose, (2) 10 mM arabinose, and (3) 25 mM arabinose with 200 ng/mL ATc.

Although the nanobody/EGFR DIII interaction specifically activated the PACE circuit, attempts to obtain active mutations using our circuit design under kinetic flow failed due to phage washout. Trial runs were conducted using 7D12 phage under the following operating conditions: (1) 1 V/h dilution rate supplemented with 25 mM arabinose (Ara) and (2) 0.5 V/h dilution rate supplemented with 10 mM arabinose. The 7D12 phage titer at both conditions dramatically dropped to 10–10^2^ pfu/mL after 24 h (Figure 6). Both titers were too low to support the generation of active mutations. Inducing gIII expression of DP with ATc seems to be the only option that allows active mutations to exist. We added a high concentration of ATc (200 ng/mL) to the first condition to guarantee PACE could proceed properly. Surprisingly, the results were controversial compared to those obtained from the T7 RNAP/T3 promoter circuit because the 7D12 phages still failed to survive under standard PACE operating conditions (∼10^4^-fold reduction after 24 h). This unexpected outcome suggests the need to further reduce the stringency to sustain an enriched phage pool, supporting the accumulation of sophisticated genotypes for the desired evolutionary pathway with robust binding.

**FIGURE 7:**
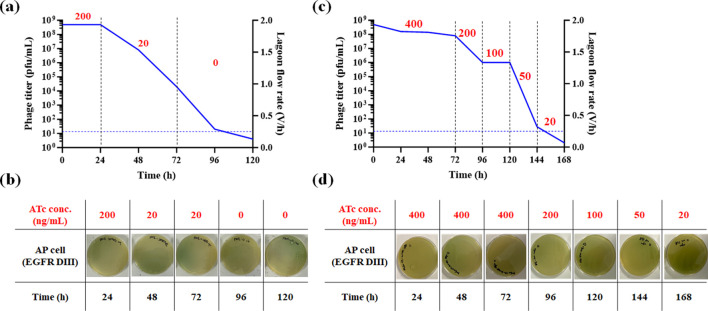
Stepwise reduction of selection stringency for 7D12 phage evolution under cytoplasmic PACE. **(a)** Phage titer over time and **(b)** corresponding plaque assay analysis under a three-step drift selection: 200 ng/mL ATc drift for 24 h, 20 ng/mL ATc drift for 48 h, and a final 48 h selection without ATc. **(c)** Phage titer over time and **(d)** corresponding plaque assay analysis under extended drift conditions with gradually decreasing ATc concentrations: 400 ng/mL (72 h), 200 ng/mL (24 h), 100 ng/mL (24 h), 50 ng/mL (24 h), and 20 ng/mL (24 h). The red number on the phage titer plots indicates the ATc concentration during that step.

To further mitigate the selection stringency, we adopted a stepwise adaptation strategy: (1) 200 ng/mL ATc drift for 24 h, (2) 20 ng/mL ATc drift for 48 h, and (3) a final 48 h selection without ATc supplementation. Except for the step ATc reduction, we also adjusted the dilution rate to 1/4 V/h to maximize phage survival. The phage titer over time is documented in Figure 7a. Although the phage titer eventually collapsed, phage titer persisted at a high concentration of ATc due to circuit-independent gIII expression from DP. The titer log plot also shows a more minor reduction in titer at the later time interval, which could be due to the phages carrying weak activity. Unfortunately, we could not isolate these phages carrying mutations with noticeable activity as a seed population for further experiments (Figure 7b). Our circuit requires a larger number of mutations to support effective residue mutations and folded structures than those previously reported. We suspect that this failure is due to the lack of a diverse high-throughput variant library. Therefore, we further extended the drift phase along by increasing the ATc concentration as follows: 400 ng/mL ATc drift for 72 h, 200 ng/mL ATc drift for 24 h, 100 ng/mL ATc drift for 24 h, 50 ng/mL ATc drift for 24 h, and 20 ng/mL ATc drift for 24 h. The phage titers over time are illustrated in Figure 7c. Despite the phage titer remaining stable at 400-100 ng/mL of ATc drift, it suddenly collapsed at the latter two steps. To confirm the activity of evolved phages, we plated the phage harvested from each step on AP cells carrying EGFR DIII. Unusually, no plaques were found in any plates after such a long period of drift, as shown in Figure 7d. Even though this result confirms that the kinetic flow prohibits gIII recombination, the plates also indicate that the occurrence of dominant mutations remains restricted. Protein affinity appears to be relevant only to circuit activation. The evolutionary potential of mammalian proteins using cytoplasmic PACE should be influenced by other factors.

## 4 Discussion

Phage-assisted continuous evolution for refining nanobody affinity poses a fundamental challenge due to the incompatibility of mammalian-originated proteins with bacterial expression systems. In this study, we demonstrated that nanobodies and EGFR DIII expressed from the bacterial cytoplasm retained a soluble, binding-competent fraction (Supplementary Figures S1, S2a,b). This indicates that complete misfolding or aggregation of nanobodies and EGFR DIII is unlikely. Both unfolded 7D12 and R30A nanobodies effectively and specifically engaged EGFR DIII after phage infection (Figure 1c). The precise interaction guided the reconstitution of the RNAP ω-subunit and further gIII transcription. Phage propagation provides compelling evidence that mammalian proteins with complex features can be adapted for two-hybrid circuit. However, no dominant adaptive mutations were identified during PANCE or PACE despite reliable circuit activation (Figures 3a, 5b, 6, 7a,c). This suggests that the POI with start-binding capability is crucial only for activating the circuit, not for shaping the evolutionary landscape.

In previous two-hybrid circuits (Supplementary Table S2), binding interactions were sufficient to drive circuit activation and dominant adaptive evolution when POI was compatible with the evolutionary context (Bardran et al., 2016; Pu et al., 2018; Zhou et al., 2024). On the contrary, mammalian proteins impose additional constraints on the cytoplasmic evolution. Although Morrison et al. successfully developed a periplasmic circuit to overcome this challenge, the confined periplasmic space imposes geometric constraints on circuit design, such as small peptides or minimal binding motifs. Therefore, they observed that the evolutionary landscape was constrained to either G200V in the V_H_ or H91Y in the V_L_ of the Her2 scFv (Morrison et al., 2021). This is the first report of restricted evolution due to limited surface area of peptides, revealing a critical factor driving the development of evolutionary trajectories.

Frequent phage titer collapse under stringent periplasmic circuit constraints has motivated the transition of PACE systems toward cytoplasmic evolution. However, the interface effect plays a vital role in evolutionary development in this context. Notably, the PD1 protein used by Ye et al. included a signal peptide, a N-loop, and the entire intracellular domain. Although the authors did not discuss these features, structural inspection revealed flexible and extended N- and C-terminal loops (Supplementary Figure S4a). This unusual extended PD1 led to the mutations initially occurring on the N-terminal signal peptide and loop. The following active mutations were established on the opposite β-sheet of the original contact surface, as illustrated in Supplementary Figure S4b (Zak et al., 2015). Even though PD1 and PDL1 lost their disulfide bonds, the increased surface area compensated for the loss of circuit activity caused by the unfolded structures and enabled convergent mutations that improve binding. This finding suggests that the PACE evolutionary landscape may depend, in part, on the binding interface geometry, area, and its accessibility rather than on protein interaction alone.

We further examine whether interface geometry could limit evolutionary accessibility in our system. The protruding loop conformation of CDR3 in nanobodies has been reported to localize most interactions with EGFR DIII to a narrow, isolated interface (CDR3) (Schmitz et al., 2013; Bannas et al., 2017) (Supplementary Figure S5). Although this CDR3-dominated interaction confers robustness to EGFR variants (Tintelnot et al., 2019), it is similar to protein/peptide interactions that limit evolutionary trajectories (Morrison et al., 2021). Since most binding sites are located in CDR3, mutagenesis in this region likely disrupts the binding of the original residues and reduces their affinity. We performed site-saturation mutagenesis of CDR3 residues within 5 Å of the interface (Supplementary Figure S6a). The predicted ΔΔG values from mutagenesis analysis show qualitative agreement with the relative binding signals measured by indirect ELISA for 5 in-house mutated variants (Supplementary Figure S6b,c). Despite that these ELISA measurements represent relative endpoint binding signals rather than quantitative kinetic affinities, they are consistent with the affinity ordering of 7D12 and R30A nanobodies. Differences in expression environment may affect protein folding and local conformational dynamics. The preservation of relative binding trends across variants suggests that the underlying interaction interface is functionally comparable at the level relevant to mutational effects. This provides qualitative support for the consistency of mutational trends.

Mutational scanning based on ΔΔG predictions indicates that approximately 80% of CDR3 substitutions are silent mutations or cause significant binding disruptions. Randomized mutations in the conserved region also destabilize the structure. Quantitative analysis of the 7D12 nanobody/EGFR DIII interface using PDBePISA is consistent with a structural constraint model (Krissinel and Henrick, 2007). Contact analysis revealed that CDR3 accounts for around 65% of interface contacts, whereas CDR1 contributes relatively fewer interactions. This interaction asymmetry concentrates binding energy within a narrow loop region, which may limit the number of positions where mutations can improve affinity without disrupting existing contacts. These observations are consistent with a model in which interface geometry may restrict mutational accessibility, even though they did not establish this as a sole determinant of the observed evolutionary result. Our findings suggest that 7D12 nanobodies may require coordinated or cooperative mutations to improve mutagenesis accessibility to surrounding residues.

Despite these structural constraints, we also considered the possibility that insufficient mutational mutagenesis may contribute to the lack of dominant adaptive mutations. Cytoplasmic or periplasmic expression always contains different levels of insoluble or misfolded protein fractions. According to our results, unfavorable portions did not disrupt phage propagation or mutational exploration. We also applied graded selection pressures and a long drift at high ATc concentration for 120 h to maintain an abundant mutant library (Supplementary Figure S7). The MP6 plasmid allows 2-3 substitutions per kbp in a single generation (Miller et al., 2020). The expected number of substitutions in our nanobody-rpoZ fusion is 1.64 per gene per generation. Since DP6 decoupled the circuit selection, mutations accumulated through immediate phage infection. Considering the short phage life cycle and the phage titer in the lagoon, the theoretical maximum of total substitutions was ∼3 × 10^7^ subs. Although single-mutated and simple combinatorial variants are expected to arise under this drift condition, we cannot exclude the possibility that complex or cooperative adaptive mutations require longer evolutionary time or are not sufficiently enriched to become detectable within the sampled population. Moreover, rare or cooperative adaptive intermediates may not reach sufficient abundance to be detected by standard plaque isolation methods, because these methods cannot exhaustively sample the phage population within the lagoon. However, given that phage propagation remained sustained without collapse or recombination over extended drift, the absence of dominant adaptive variants suggests that the results are not readily explained by incomplete exploration alone. Incomplete sampling and cooperative mutational requirements remain plausible contributors. These factors may act in combination; therefore, it is difficult to assign a single dominant cause to the observed lack of evolvability.

Other factors may contribute to the observed constraint on adaptive mutations. The loss of intramolecular disulfide bonds and glycosylation within nanobodies and EGFR DIII introduced an additional layer of interaction variability. Without the stabilization provided by glycosylation and disulfide bonds, freely fluctuating loops could lead to less well-defined or transient binding contacts. Further, cytoplasmic expression of mammalian proteins usually comprises a higher proportion of misfolded and insoluble proteins. Both defects increase the difficulty in mutational convergence toward improved affinity states during PACE due to the variability in binding interactions. However, these effects should be minor since each phage propagation was achieved under functional nanobody/EGFR DIII engagement. These results indicate that binding competence and circuit activation were preserved. Instead, such factors likely act in combination with limitations in mutational accessibility.

Our findings highlight the mechanistic distinction between circuit activation and evolvability. In this context, alternative starting binders and affinity-matured variants probe different regions of the evolutionary landscape due to their distinct accessibility profiles at their interfaces. Their starting points may enable the emergence of adaptive mutations; however, such alternatives do not contradict the structural constraints on the native interface of 7D12 nanobodies. Instead, they emphasize the dependence of evolvability on interface accessibility. The evolution using other binders or engineered starting variants would be valuable for evaluating the generality of this limitation across different protein–protein interfaces. Overall, these observations decouple the commonly assumed relationship between successful circuit activation and efficient adaptive evolution. They suggest that the structural accessibility of beneficial mutations plays a major role in shaping evolvability in cytoplasmic PACE. Additional factors, such as structural stability and cytoplasmic expression level, may further limit the efficiency of evolutionary convergence.

## 5 Conclusion

In this study, we investigated cytoplasmic PANCE and PACE using the 7D12 nanobody/EGFR DIII system to evaluate whether single-domain mammalian proteins can support circuit activation and affinity evolution. This system enabled robust circuit activation and sustained phage propagation. However, no detectable adaptive mutations were observed under the conditions tested despite extended mutagenesis designed to increase sequence diversity.

These results highlight that successful circuit activation does not necessarily imply evolvability. The absence of evolutionary convergence is most consistent with a contribution from structural constraints at the binding interface, even though this interpretation cannot distinguish between intrinsic structural inaccessibility or local fitness landscape effects. Other factors associated with the cytoplasmic expression environment may also act in combination with the structurally constrained interface, further limiting the efficiency of evolutionary convergence.

These limitations suggest that alternative strategies may be required to explore broader mutational pathways while improving organismal compatibility. Recent advances in artificial intelligence (AI) protein design, such as ProteinMPNN (Dauparas et al., 2022) and RFdiffusion (Watson et al., 2023), have enabled the generation of alternative binders, including anti-EGFR (Cao et al., 2022; Pacesa et al., 2025) variants that do not rely on intramolecular disulfide bonds. Such approaches may facilitate access to diverse mutational trajectories while alleviating constraints imposed by organismal incompatibility.

Although this study remains system-specific, it still suggests that the limitations observed in PACE arise from the interplay between interface accessibility and organism-level constraints. Recognizing this interplay is important for evaluating whether similar constraints apply to other mammalian protein systems.

## Data Availability

The datasets presented in this study can be found in online repositories. The names of the repository/repositories and accession number(s) can be found in the article/Supplementary Material.
