# Supplementary material for: Dissecting the disconnect between circuit activation and dominant adaptive evolution in cytoplasmic phage-assisted continuous evolution (PACE) of an EGFR nanobody
Source: Front Bioeng Biotechnol. 2026 May 29;14:1829373. doi: 10.3389/fbioe.2026.1829373 (PMC13260496; doi:10.3389/fbioe.2026.1829373)
Supplement: Supplementary file 1 [file Supplementaryfile1.docx]

**Supplementary**

**Dissecting the disconnect between circuit activation and adaptive evolution in cytoplasmic phage-assisted continuous evolution (PACE)**

*Jie-Ning Chuang^1^, Jacob Purcell^2^, Loki Sangalli^2^, Joseph (Sefi) Rosenbluh^2^, Gavin J. Knott^2,3^, Simon Corrie^1^, Gil Garnier^1^**

1 Bioresource Processing Research Institute of Australia (BioPRIA), Department of Chemical and Biological Engineering, Monash University, Clayton, Victoria 3800, Australia

2 Department of Biochemistry and Molecular Biology, Biomedicine Discovery Institute, Monash University, Clayton 3800, Australia

3 AI Protein Design Program, Biomedicine Discovery Institute, Monash University, VIC 3800, Australia

*Correspondence:

Corresponding Author

[gil.garnier@monash.edu](mailto:gil.garnier@monash.edu)

Bioresource Processing Research Institute of Australia (BioPRIA), Department of Chemical and Biological Engineering, Monash University, Clayton, Victoria 3800, Australia


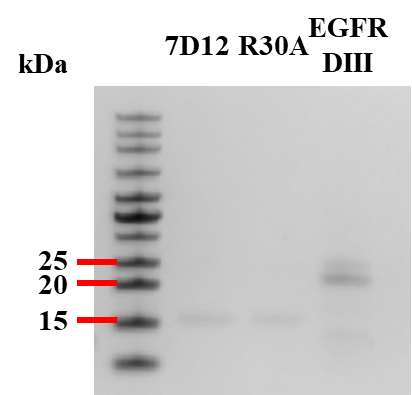


**Figure S1**. SDS-PAGE analysis of 7D12, R30A, and EGFR DIII proteins expressed from BL21(DE3).


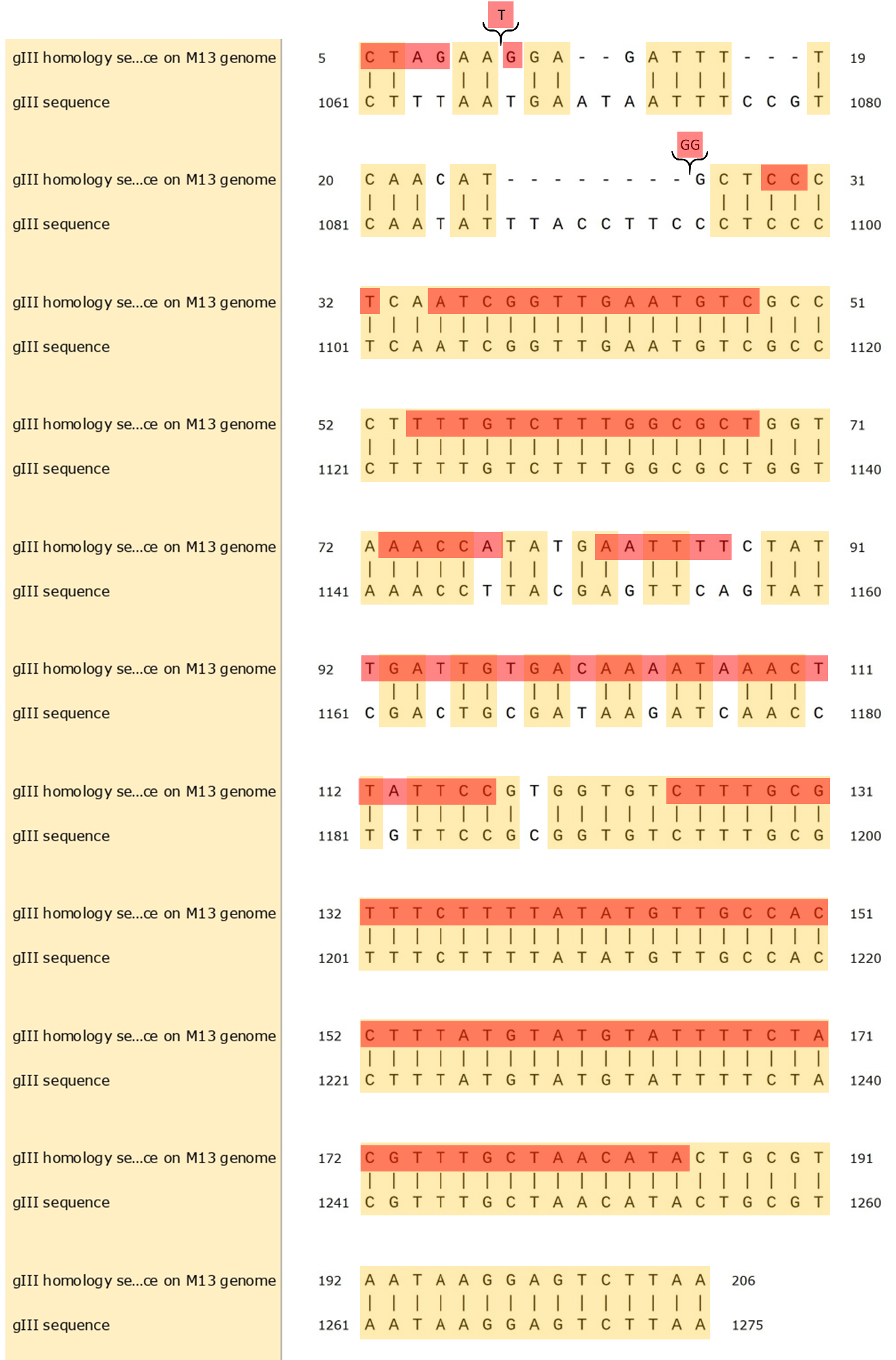


**Figure S2**. Sequence alignment of the gIII homology region on the M13 phage genome and the wild-type gIII sequence. Identical nucleotides between the two sequences are highlighted in yellow. Regions shown in red indicate sequence modifications (deletions and insertions) introduced in the updated plasmid design from David Liu’s group to reduce homologous recombination of gIII [Zhang et al., 2024].

**Table S1**. Kinetic parameters of 7D12 and R30A nanobody in complex with EGFR DIII measured by BLI.

|  | ***k_on_* (1/Ms)** | ***k_off_* (1/s)** | ***K_D_* (nM)** |
| --- | --- | --- | --- |
| **7D12:EGFR DIII** | 2.1×10^3^ | 9.6×10^-5^ | 47 |
| **R30A:EGFR DIII** | 5.7×10^3^ | 8.5×10^-4^ | 149 |


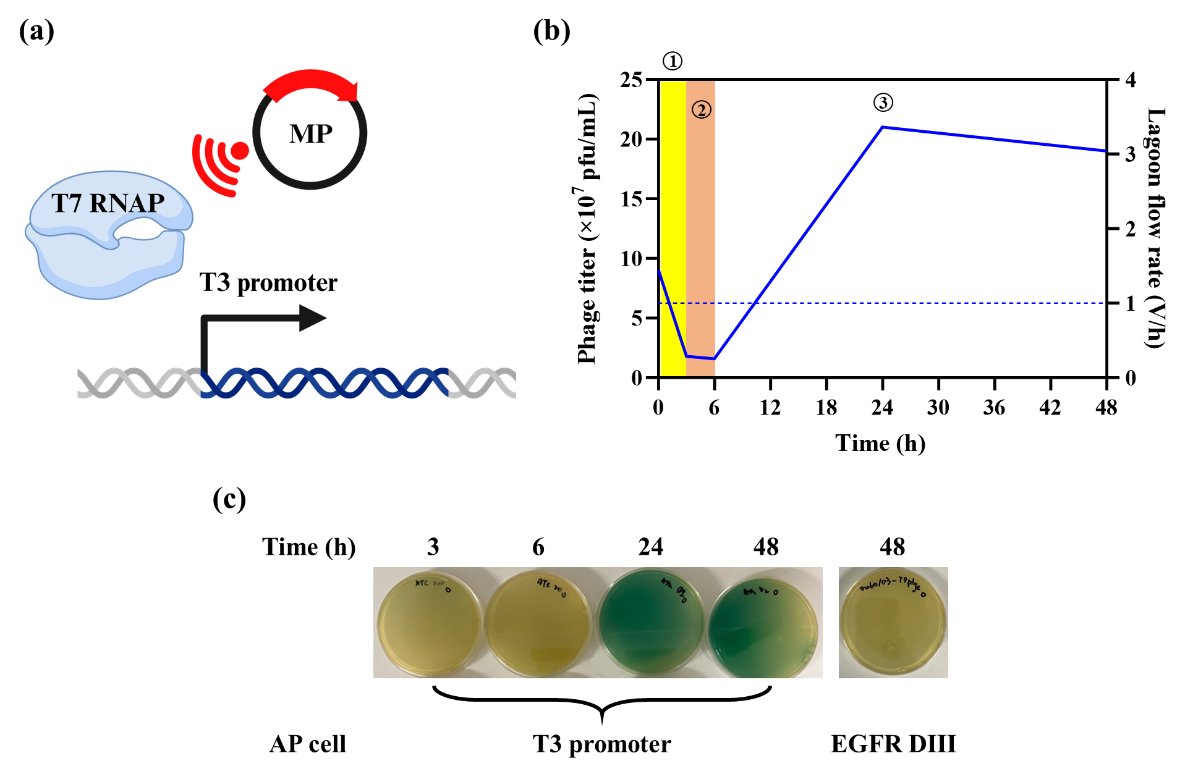


**Figure S3**. (**a**) Circuit design of evolving T7 RNAP to recognize T3 promoter. (**b**) T7 RNAP phage titer and lagoon flow rate over time under different ATc supplementation strategies of PACE: (1) 100 ng/mL ATc, (2) 10 ng/mL ATc, and (3) no ATc supplementation. (**c**) Plaque assay analysis of evolved T7 RNAP phage after 3, 6, 24, and 48 h of PACE selection using AP cells carrying T3 promoter and EGFR DIII.


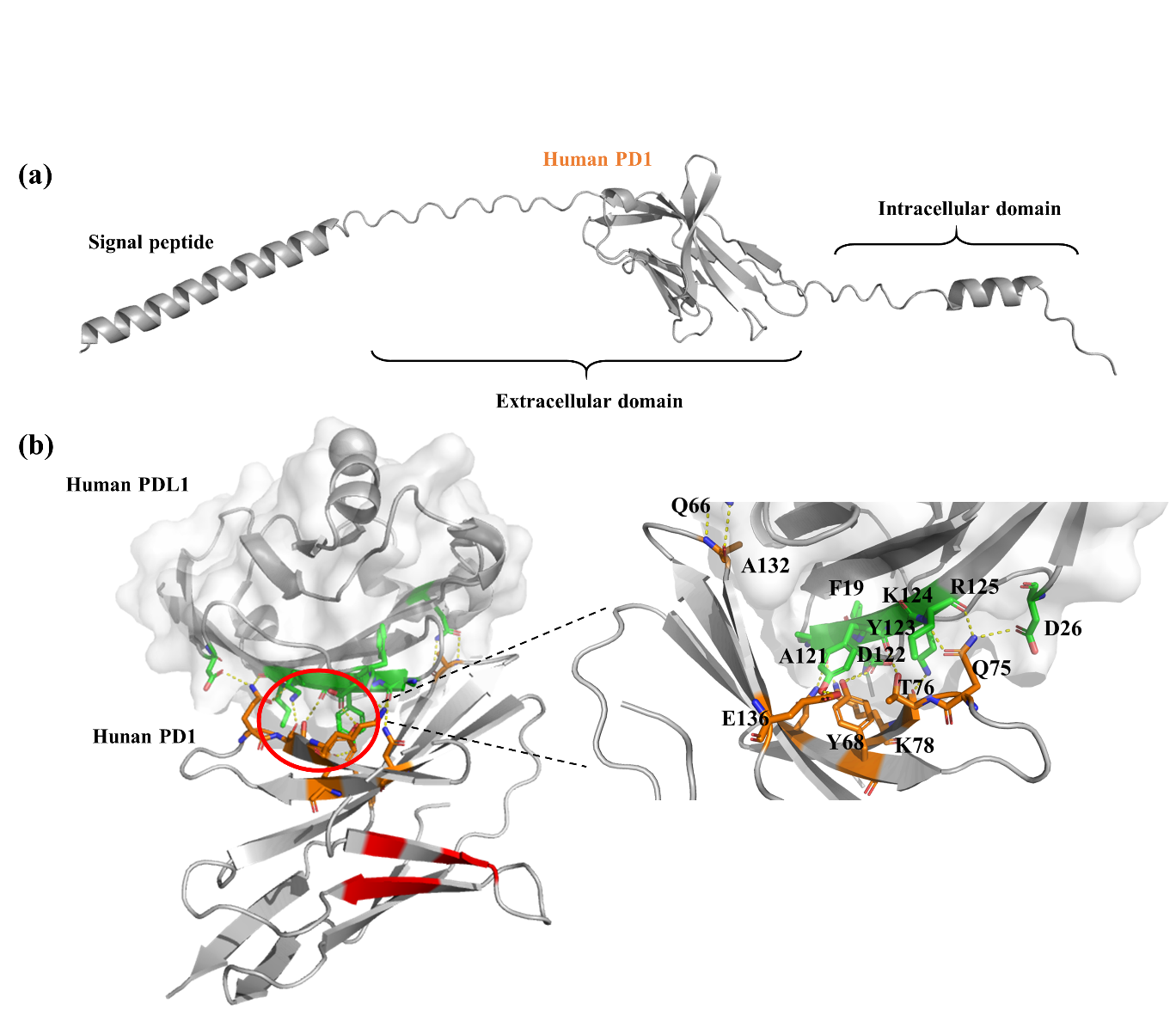


**Figure S4**. Structural analysis of full-length human PD1 and human PD1 in complex with human PDL1 [Zak et al, 2015]. (**a**) Alphafold-predicted structure of human PD1 with extra and intracellular domains [Jumper et al, 2021] (**b**) Crystalized structure of PDB: 4ZQK and the targeting residues between PD1 (orange) and PDL1 (green). Red residues in both panels represent the mutated sites reported in Ye et al. [Ye et al, 2020].


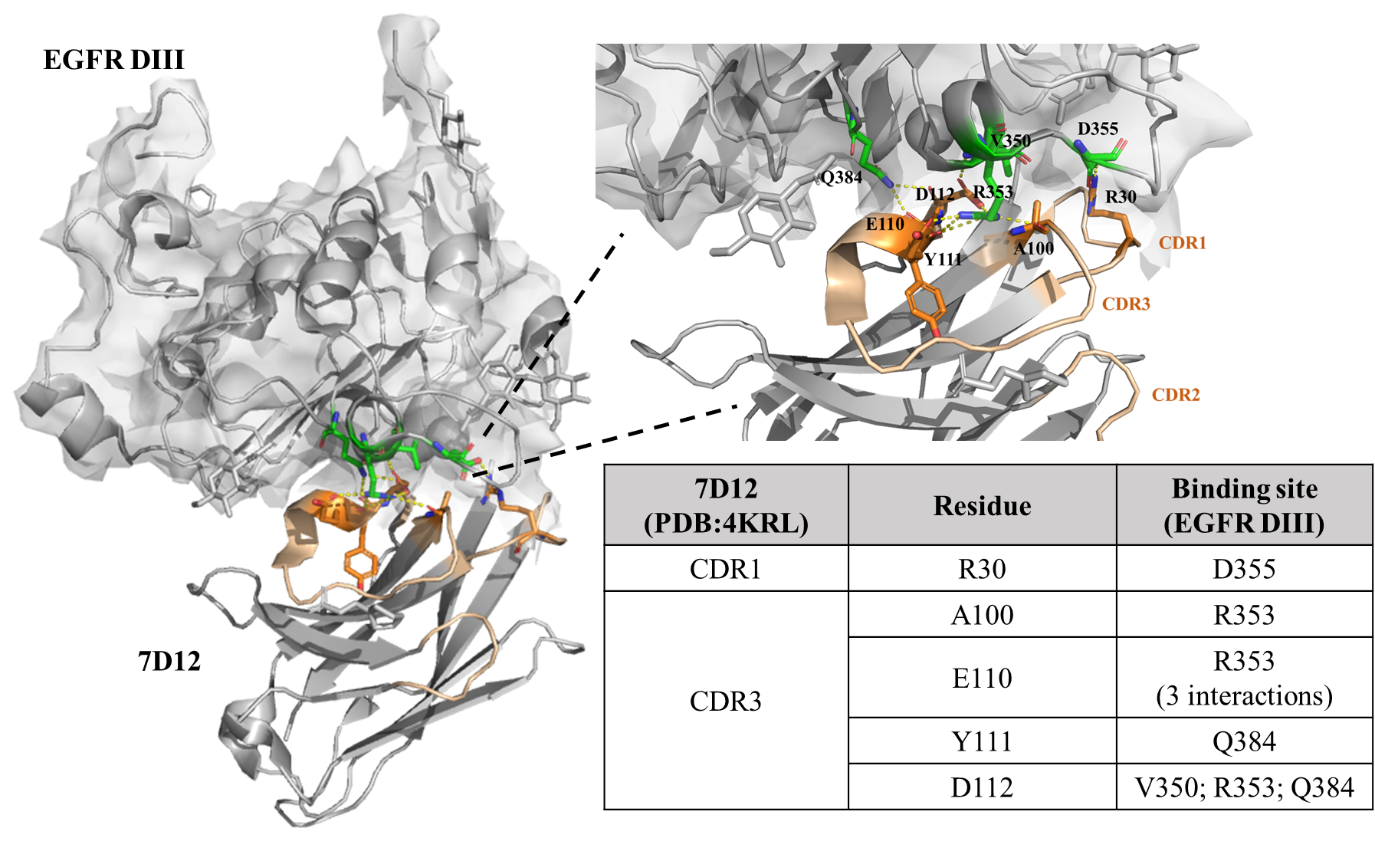


**Figure S5**. Structural analysis of 7D12 nanobody in complex with EGFR DIII [Schmitz et al, 2013]. All CDRs are colored in light orange. Close-up of 7D12:EGFR DIII interface showing the contact residues of the nanobody (dark orange) interacting with EGFR DIII (green).

**Table S2**. Summary of PACE using a two-hybrid circuit.


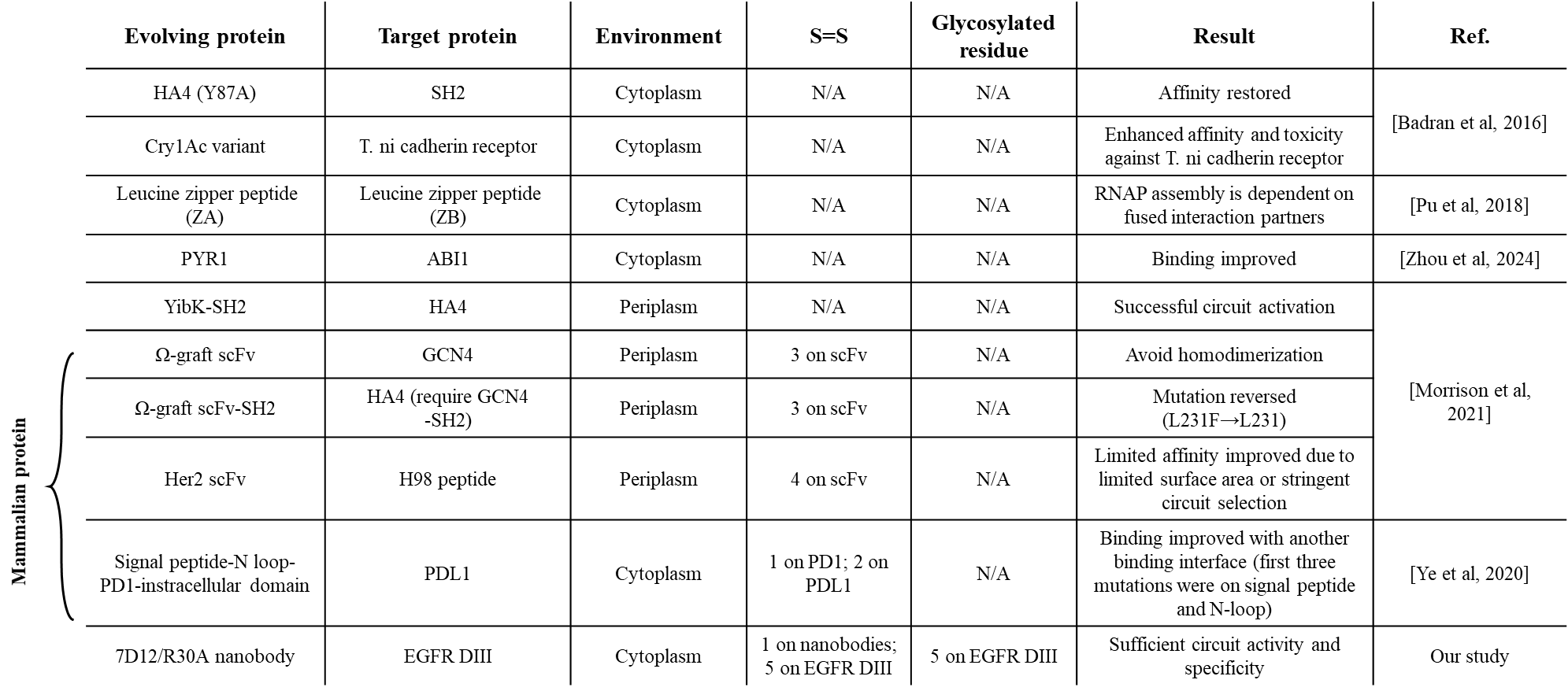


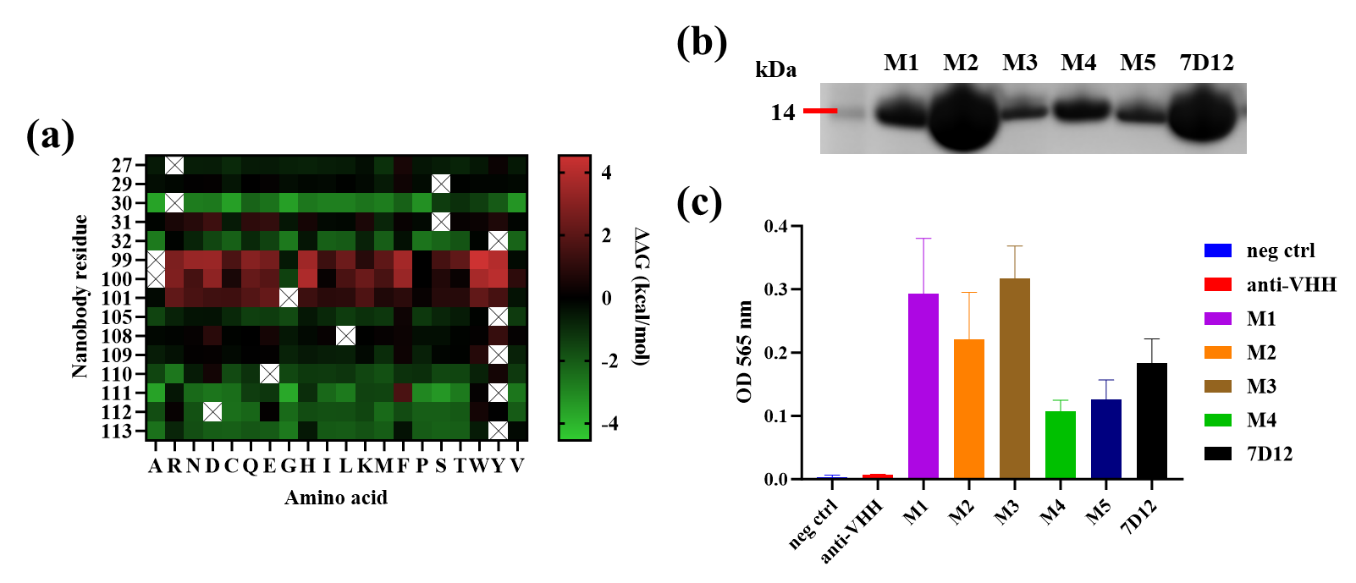


**Figure S6**. (a) Site-saturation scanning of residues located within the CDR1 and CDR3 regions of the 7D12 nanobody. (b) Representative SDS–PAGE showing soluble expression of selected mutants: M1 (G101E), M2 (A100E), M3 (A99W/A100Y), M4 (R30A), M5 (R30A/D112A), and the 7D12 nanobody (wild-type). (c) Binding responses of the mutants and WT nanobody toward EGFR DIII measured by indirect ELISA. Each value represents the mean ± SD from three independent replicates.


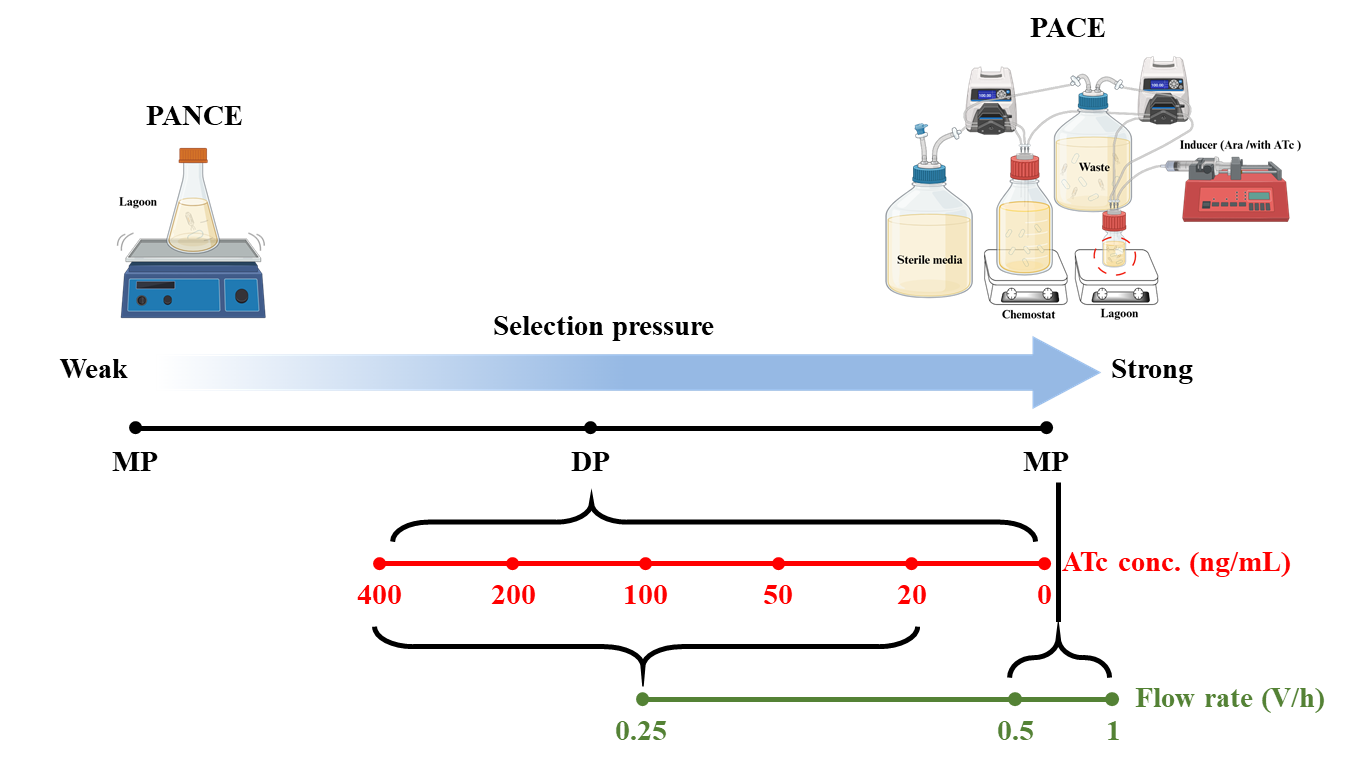


**Figure S7**. Strategy selected for graded selection pressure during cytoplasmic PACE.

**References**

1. Zhang, E., Neugebauer, M.E., Krasnow, N.A. and Liu, D.R. (2024). Phage-assisted evolution of highly active cytosine base editors with enhanced selectivity and minimal sequence context preference. *Nat. Commun.* 15 (1), 1697. doi:10.1038/s41467-024-45969-7.
2. Zak, K.M., Kitel, R., Przetocka, S., Golik, P., Guzik, K., Musielak, B., et al. (2015). Structure of the complex of human programmed death 1, PD-1, and its ligand PD-L1. *Structure*. 23 (12), 2341-2348. doi:10.1016/j.str.2015.09.010.
3. Jumper, J., Evans, R., Pritzel, A., Green, T., Figurnov, M., Ronneberger, O., et al. (2021). Highly accurate protein structure prediction with AlphaFold. *Nature*. 596 (7873), 583-589. doi:10.1038/s41586-021-03819-2.
4. Ye, X., Tu, M., Piao, M., Yang, L., Zhou, Z., Li, Z., et al. (2020). Using phage-assisted continuous evolution (PACE) to evolve human PD1. *Exp. Cell Res.* 396 (1), 112244. doi:10.1016/j.yexcr.2020.112244.
5. Schmitz, Karl R., Bagchi, A., Roovers, Rob C., van Bergen en Henegouwen, Paul M.P. and Ferguson, Kathryn M. (2013). Structural evaluation of EGFR inhibition mechanisms for nanobodies/VHH domains. *Structure*. 21 (7), 1214-1224. doi:10.1016/j.str.2013.05.008.
6. Bardran, A.H., Guzov, V.M., Huai, Q., Kemp, M.M., Vishwanath, P., Kain, W., et al. (2016). Continuous evolution of Bacillus thuringiensis toxins overcomes insect resistance. *Nature*. 533 (7601), 58-63. doi:10.1038/nature17938.
7. Pu, J., Zinkus-Boltz, J. and Dickinson, B.C. (2018). Evolution of a split RNA polymerase as a versatile biosensor platform. *Nat. Chem. Biol.* 13 (4), 432-438. doi:10.1038/nchembio.2299.
8. Zhou, Z., Wang, Y.-Q., Zheng, X.-N., Zhang, X.-H., Ji, L.-Y., Han, J.-Y., et al. (2024). Optimizing ABA-based chemically induced proximity for enhanced intracellular transcriptional activation and modification response to ABA. *Sci. China Life Sci.* 67 (12), 2650-2663. doi:10.1007/s11427-024-2707-9.
9. Morrison, M.S., Wang, T., Raguram, A., Hemez, C. and Liu, D.R. (2021). Disulfide-compatible phage-assisted continuous evolution in the periplasmic space. *Nat. Commun.* 12 (1), 5959. doi:10.1038/s41467-021-26279-8.
